# The characteristics and prognosis of patients fulfilling the Appropriateness Evaluation Protocol in a medical admission unit; a prospective observational study

**DOI:** 10.1186/1472-6963-11-152

**Published:** 2011-06-27

**Authors:** Mikkel Brabrand, Torben Knudsen, Jesper Hallas

**Affiliations:** 1 Department of Medicine, Sydvestjysk Sygehus Esbjerg, Finsensgade 35, DK-6700 Esbjerg, Denmark; 2 Department of Clinical Pharmacology, University of Southern Denmark, J.B. Winsløws Vej 19, DK-5000 Odense C, Denmark

## Abstract

**Background:**

To examine the prognostic significance of fulfilling at least one of the Appropriateness Evaluation Protocol (AEP) criteria.

**Methods:**

Prospective observational cohort study at medical admission units at a regional teaching hospital in Denmark. 3,050 consecutively admitted patients were included, median age 66 (IQR: 50-77), 48% female.

We assessed the fulfilment of the AEP criteria and mortality data, length of stay, readmissions and co-morbidity. We analyzed the association between day of admission and time of day and compared the opinion of the admitting doctors and nurses on the relevancy of admission.

**Results:**

61.9% of the patients fulfilled the AEP criteria. Patients fulfilling were older (p < 0.001), had a higher in-hospital mortality (p < 0.001), a higher 30-days mortality (p < 0.001), a longer length of stay (p < 0.001), more readmissions within 30 days (p < 0.001) and higher co-morbidity (p < 0.001). There were no association between day of admission and fulfilment of AEP criteria, but significantly fewer patients fulfilled the AEP criteria in the morning hours (p < 0.05). The nurses found 79.1% of the admissions relevant with a sensitivity of 84.8% and a specificity of 30.1% with a Kappa of 0.16. The doctors found 76.2% of the admissions relevant with a sensitivity of 86.4% and a specificity of 40.9% and a Kappa of 0.29.

**Conclusions:**

Fulfilment of the AEP criteria adequately reflect increased morbidity and mortality of acutely admitted medical patients.

## Background

Most admissions to medical departments are unplanned. At the same time, the annual number of admissions is rising steadily and the number of available beds is falling. Usage of the medical inpatient beds therefore has to be as effective as possible and as many patients as possible need to be referred for treatment as out-patients or in primary care.

In 1981, Gertman and Restuccia introduced the Appropriateness Evaluation Protocol (AEP). There were two versions meant as instruments to review whether an acute admission or whether an in-patient day was spent appropriately[1]. It was designed to be diagnosis independent for patients in adult medicine, surgery and gynaecology. The criteria for reviewing admission were based on objective measures of severity of disease and required level of care (e.g. electrolyte abnormality, need for surgery and intravenous treatment, see table 1). If just one of the individual 16 criteria was met, the admission was designated as appropriate. The AEP includes an override option where raters can override inappropriateness if so deemed. AEP has been used to evaluate populations in the United States of America and in many European countries and a European version has been implemented[2]. AEP is generally accepted as a good tool of reviewing the appropriateness of admissions[2].

**Table 1:** Fulfilment of the Appropriateness Evaluation Protocol criteria by 3,050 subjects admitted to the medical admission units

	n (%)
**Surgery or other procedure in 24 hours requiring**	12 (0.4%)
**1. General/regional anaesthesia; and/or**	
**2. Equipment or other facilities only for inpatients**	
**Vital signs monitoring at least every 2 hours**	336 (11.0%)
**Intravenous medication and/or fluid replacement**	1,451 (47.6%)
**- medication**	954 (31.3%)
**- fluid replacement**	497 (16.3%)
**Observation for toxic reaction to medication**	225 (7.4%)
**Continuous or intermittent (at least every 8 hours) respiratory assistance**	178 (5.8%)
**Severe electrolyte or blood gas abnormality**	381 (12.5%)
**Acute loss of sight or hearing**	0 (0.0%)
**Acute loss of ability to move any body part**	0 (0.0%)
**Persistent fever > 38.0°C for more than 5 days**	98 (3.2%)
**Active bleeding**	2 (0.1%)
**Wound dehiscence or evisceration**	0 (0.0%)
**Pulse rate**	96 (3.1%)
**Blood pressure**	363 (11.9%)
**Sudden onset of unconsciousness**	117 (3.8%)
**ECG evidence of acute ischemia**	92 (3.0%)

**Total**	**1,889 (61.9%)**

Most reports on AEP have focused on the proportion of patients fulfilling the AEP criteria at various admission units throughout the World. Our aim is therefore to examine the prognostic significance of fulfilling at least one of the AEP criteria.

## Methods

### Setting

Sydvestjysk Sygehus Esbjerg is a 460 bed regional hospital in the western part of Denmark with a contingency population of approximately 220,000. All subspecialties of internal medicine are represented. There is an eight bed level 2 intensive care unit along with general surgery, orthopaedics, ear- nose and throat diseases, neurology and paediatrics.

Patients can be admitted to the medical admission units by their general practitioner, emergency medical service, out-patient clinics, emergency department and ambulance services. The medical admission units are regarded as all other medical departments in the hospital and are not designated as part of the emergency department. Patients are thus always admitted to a medical admission unit but can then be discharged from the admission unit (like any other medical department) or transferred to other medical departments. There is a 24-hour time limit on admissions in the admission units, and patients requiring admissions lasting longer than this must be transferred to other departments. Sydvestjysk Sygehus Esbjerg has two medical admission units (MAU), one for general internal medicine and one for cardiology. Both departments are staffed by an intern and an attending physician around the clock. The departments also share a resident in internal medicine or family medicine who covers both sections.

### Design and data

We conducted a prospective observational cohort study of all patients admitted through the medical admission units at our hospital. All patients admitted from October 2nd 2008 until February 19th 2009 were included in the study. A sample size calculation was not conducted for this part of our study. The sample size was based on the development of a risk stratification study not yet published.

Upon admission (and usually within 15 minutes) a nurse recorded the vital signs and registered these along with the reason for admission (suspected diagnose) on a form. The admitting doctor, after interviewing and examining the patient (within one or two hours after admission), completed a form with information on the history of the patient, their physical examination results, an analysis of the EKG (if so ordered) and if the patient needed checking of the vital signs at least every two hours. The nurse and doctor were asked if they found that the patient was in need of admission to a medical department, and was thus asked to make this decision within a few hours after the patient arrived at the department and before any laboratory results were known. Senior physicians decided the final treatment of the patients and the admitting doctors completing the forms for this study had no influence on this. After discharge one of the authors (MB) completed a chart review of the admission notes but not subsequent notes from the admission. After inclusion of all patients, we extracted blood test results and treatments registered in the hospital computer systems.

In case of incomplete filling of the forms, we sought the information in the charts and/or the nurse's notes. We were unable to locate data on fever in more than five days in 166 patients (5.4%), heart rate in nine patients (0.3%) and blood pressure in seven patients (0.2%). We were not able to ascertain if there were signs of acute ischemia on the EKG in 1,180 (38.7%) patients but not all patients had an EKG taken and we were not able to differentiate between these. According to the European version of AEP, telemetry is not automatically fulfilment of an AEP criterion[2].

We used the Danish translation of the AEP criteria introduced by Ishøy et al.[3] and only present data on the objective part of the AEP criteria. There were no analyses on interobserver agreement in our cohort.

All data were checked both by MB and a secretary. The study was approved by the Danish Data Protection Agency. Approval by the regional Ethics Committee was not required by Danish law.

### Statistics

Data are reported as median (Inter Quartile Range (IQR)) or proportions wherever appropriate. χ^2 ^test or Wilcoxon Rank-Sum test are used to test differences. We calculated the sensitivity, specificity, positive predictive value and negative predictive value of AEP fulfilment by using as gold standard the fulfilment of the AEP criteria. The Kappa value of agreement of nurses and doctors with the AEP criteria was calculated using Cohen's Kappa. The association between AEP and in-hospital mortality was examined using χ^2 ^test. The survival of patients was examined using a Kaplan-Meyer plot. Difference in survival was tested using the Log-Rank test. STATA version 10.1 (StataCorp, College Station, TX, USA) was used for analyses.

## Results

A total of 3,050 patients were included in our study, 48.0% female and median age 66 years (IQR: 50-77). A total of 84 (2.8%) patients died during their admission. The criteria most often fulfilled were need of parenteral treatment (47.6%), electrolyte or blood gas abnormality (12.5%), blood pressure abnormalities (11.9%) and need for checking of vital signs at least every two hours (11.0%), see table 1. We found that 67.7% of the patients admitted to the general internal MAU fulfilled at least one AEP criterion, compared to 51.1% of the patients admitted to the cardiology MAU (p < 0.001). If we included telemetry as an AEP criteria (as in the original report[1], but which is not part of the European version of AEP[2]), 2,180 patients (71.5%) fulfilled the AEP criteria, 77.8% at the cardiologic MAU and 68.0% at the general MAU (p < 0.001).

Differences between patients fulfilling at least one AEP criterion, including Charlson co-morbidity score, or none can be seen in table 2.

**Table 2:** Differences of patients fulfilling the Appropriateness Evaluation Protocol (AEP) criteria

	AEP compliant (n = 1,889)	AEP non-compliant (n = 1,161)	Test
Age	68 (53-79) years	63 (48-74)	p < 0.001
Gender (female)	49.1%	46.2%	p = 0.12
Mortality	3.9%	0.9%	p < 0.001
Mortality 30 days after discharge	9.8%	2.8%	p < 0.001
Length of stay	50.0 (16.3-148.0) hours	18.5 (7.6-51.0) hours	p < 0.001
Length of stay more than 48 hours (n = 1,291, 42.3%)	75.5%	52.0%	p < 0.001
Need for specialized services not included in the AEP criteria (e.g. pacemaker implementation, haemodialysis, coloscopi)	7.2%	3.7%	p < 0.001
Readmission within 30 days after discharge	21.7%	16.0%	p < 0.001
Readmission within 30 days after discharge when admitted for less than 48 hours	30.7%	29.0%	p = 0.43
Co-morbidity defined by one or more co-morbidities according to the Charlson score [10]	59.3%	51.3%	p < 0.001
Nursing home resident	5.8%	2.3%	p < 0.001

### Variation on days and hours

**Figure 1:**
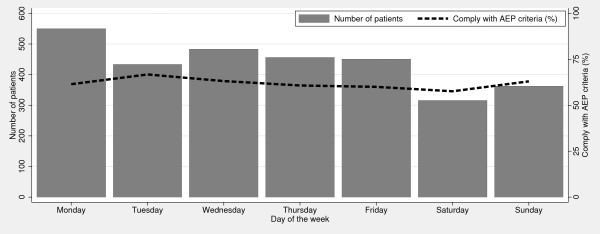
**Percentage of patients complying with the AEP criteria per day of the week**.

**Figure 2:**
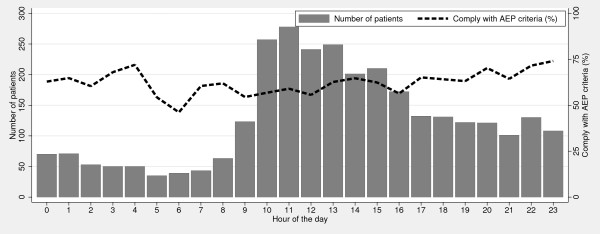
**Percentage of patients complying with the AEP criteria per hour of the day**.

We found no statistical significant differences in the percentages of patients complying with the AEP criteria according to of day of admission (p = 0.22), but a significant difference according to time of day at admission (p < 0.05), see Figures 1 and 2.

### Doctors and nurses opinion on relevancy of admission

When asking the nurses if they found the admissions relevant, they found 79.1% of the admissions relevant. Calculating the Kappa value of nurses agreeing with the AEP criteria, we found a kappa of 0.16 (p < 0.0001). When comparing their assessment with the fulfilment of the AEP criteria, we found a 66.3% agreement, with a sensitivity of 84.8%, a specificity of 30.1%, a positive predictive value of 66.3% and a negative predictive value of 54.9%. The doctors found 76.2% of the admissions relevant. Calculating the Kappa value of doctors agreeing with the AEP criteria, we found a kappa of 0.29 (p < 0.0001). We found a 71.3% agreement with AEP and a sensitivity of 86.4%, a specificity of 40.9%, a positive predictive value of 71.3% and a negative predictive value of 63.9%.

### AEP and the fate of the patients

**Figure 3:**
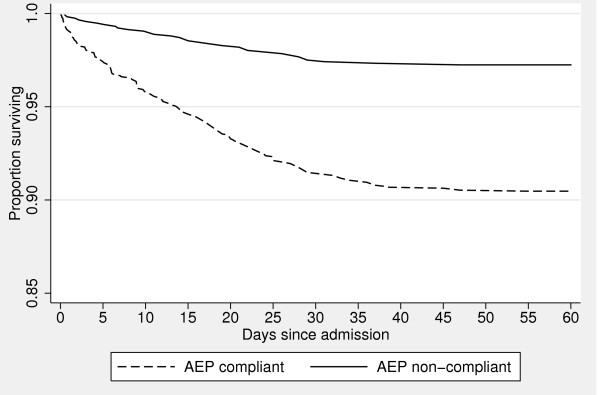
**Kaplan-Meyer plot of in-hospital mortality and Appropriateness Evaluation Protocol (AEP) fulfilment, p < 0.05**. Please note that the proportion of surviving patients start at 0.85.

Fulfilment of the AEP criteria was significantly associated with in-hospital mortality, OR 4.7 (95% CI: 2.4-9.1, p < 0.001), see Figure 3 for a Kaplan-Meyer plot illustrating the association between in-hospital mortality and AEP fulfilment. As shown in Figure 3, we find a significant association on the mortality with fulfilment of the AEP criteria.

The characteristics of the 10 patients who died and did not fulfil the AEP criteria can be seen in table 3. Their median age was 77.5 years (IQR: 69-86) and length of stay 114.0 (IQR: 41.9-161.5) hours. Seven (70.0%) had an order of do not attempt resuscitation at the time of death.

**Table 3:** Details of the 10 patients who died and did not fulfil the AEP criteria

Sex	Age	Presenting complaint	Discharge diagnosis	DNAR ordered	LOS (hours)
Male	86	Dehydration	Ileus	Yes	12.6
Female	73	Dyspnoea	COPD	Yes	234.7
Male	51	Dyspnoea	Liver failure	Yes	189.5
Male	90	Pulmonary cancer	Dyspnoea	Yes	41.9
Male	80	Cough	Pneumonia	Yes	51.3
Female	88	Dehydration	Septicaemia	Yes	102.4
Male	59	General debility	Dyspnoea	No	125.7
Male	75	Pneumonia	Pneumonia	Yes	155.3
Male	69	Syncope	Heart failure	No	161.5
Female	83	COPD	COPD	No	18.4

DNAR = Do not attempt resuscitation

Of the 1,355 patients who were discharged within 24 hours, 663 (48.9%) fulfilled the AEP criteria. Of these 663 patients, 117 (17.7%) were readmitted to a Danish hospital with 30 days after discharge (p < 0.01). Of the 663 patients fulfilling the AEP criteria, 45 patients (6.8%) died within 30 days after discharge (p < 0.001).

## Discussion

When prospectively reviewing 3,050 admissions to the two MAU's at our hospital we found that 61.9% of the patients fulfilled the AEP criteria for appropriate admission. Significantly more patients fulfilled the criteria at the general internal MAU than at the cardiologic MAU. The criteria most often fulfilled were need of parenteral treatment (47.6%), electrolyte or blood gas abnormality (12.5%), blood pressure abnormalities (11.9%) and need for checking of vital signs at least every two hours (11.0%). Patients, who fulfilled the AEP criteria were hospitalized significantly longer, more often needed specialized services and had more re-admissions within 30 days after discharge. Patients who fulfilled the AEP criteria had significantly higher in-hospital mortality as well as within 30 days after discharge. Nurses had a 66.3% agreement with the AEP criteria for appropriateness of admission while doctors had a 71.3% agreement.

The overall 61.9% fulfilment of the AEP criteria is not highly abnormal as previous reports from Denmark and Sweden have shown between 11% and 33% inappropriate admission rates when using the AEP[3-5]. Looking at the international literature our rate of non-fulfilment of AEP criteria is not as deviating, though still high. Papers from around Europe from both secondary and tertiary hospitals report non-fulfilment rates ranging from 10.9 to 31%[6-9]. One of the reasons that our proportion of patients fulfilling the AEP criteria is not higher could be due to organization. We do not, at the moment, have any alternatives to admission for patients in need of specialist care within a few days. Were this possible, it is very likely that more non-AEP compliant patients could have been managed without having to be admitted. More patients admitted to the general medical admission unit fulfilled the AEP criteria than at the cardiology admission unit with using the European version of the AEP criteria[2]. However, when using the original American version[1], the proportion of patients fulfilling the criteria at the cardiology admission rose to such an extent that significantly more patients complied in cardiology. The reason for this is probably the use of telemetry, which is not an integrated part of the European version of the AEP criteria. Most patients admitted to be evaluated for Acute Coronary Syndrome need little more than an EKG and blood tests and thus do not automatically fulfil the AEP criteria. That parenteral treatment was the most often fulfilled criterion is not surprising. Both data from Sweden[5] and Denmark[3] have shown a similar picture.

Our nurses and doctors in the MAU's found more admissions relevant than the AEP and the doctors also had a higher level of agreement (71.3%) with AEP than the nurses (66.3%). Our admitting doctors were all junior, mostly interns and a few junior residents. This could be the reason that our results differ from previous data from Denmark. In a paper from 2005 Ishøy et al. asked senior physicians to review 518 acute admissions from two hospitals. They found a sensitivity of 91%, a specificity of 79% and a positive predictive value of 95% and a negative predictive value of 62% of the AEP against a global assessment of the admission's relevance[3]. Also our doctors were asked to review the relevancy of the admission during the first few hours after arrival of the patients. This could be another explanation.

Patients fulfilling the AEP criteria seem to be more ill than the rest of the cohort. We found a longer length of stay, a higher need for specialized services (e.g. pacemaker implantation, non-invasive ventilation and haemodialysis), a higher risk of re-admission within 30 days after discharge, a higher co-morbidity measured by the Charlson index[10] and a higher in-hospital mortality and 30 days after discharge. As far as we know data like this have not been presented previously in the international literature. This is hardly surprising. The AEP criteria were designed to identify patients in need of admission. Patients admitted are obviously more ill than patients that can be managed with a lower level of care than a hospital. But a difference in in-hospital mortality from 0.9% to 3.9% (our crude mortality is 2.3%) is striking. Table 3 presents data on the patients who died and who did not fulfil the AEP criteria. Seven of the 10 patients had a decision of 'Do Not Attempt Resuscitation' (DNAR). Although death in a patient whose admission was deemed unnecessary may seem like the ultimate failure of the AEP, the high rate of DNAR decisions suggests a more complicated issue.

Our study has some limitations. First we only reviewed the admission notes and not the complete charts from all admissions. All patients admitted to our MAU's are reviewed by a senior physician (attending or senior resident) within the first 24 hours and the treatment adjusted if required. We have not reviewed their notes and thus have incomplete information on later orders on parenteral treatment. This bears the risk that our numbers are too low. Second we have incomplete data. As for pulse and blood pressure this is only a minor proportion of the patients, but we lack data on the EKG in 1,180 patients and fever in more than five days on 166. Not all patients have had an EKG taken and we are unfortunately not able to distinguish between these. In previous reports abnormality of the EKG has not been a major issue[3], and we believe this also applies to our population. Third we have asked only one nurse and one doctor to assess each patient. We therefore know nothing on interrater agreement on the fulfilment of the AEP criteria or the relevancy of admission between several members of each profession.

## Conclusions

We conclude that fulfilment of the AEP criteria adequately reflect increased morbidity and mortality of acutely admitted medical patients.

## Competing interests

The authors declare that they have no competing interests.

## Authors' contributions

MB completed the data collection and statistic calculations and drafted the manuscript. All authors participated in the design of the study. All authors read and approved the final manuscript.

## Pre-publication history

The pre-publication history for this paper can be accessed here:

http://www.biomedcentral.com/1472-6963/11/152/prepub
